# Direct fluoroscopic evidence of preserved side-branch drainage with a multi-hole covered metal stent for benign hepaticojejunostomy stricture after pancreaticoduodenectomy

**DOI:** 10.1055/a-2936-2633

**Published:** 2026-08-31

**Authors:** Eisuke Ozawa, Kosuke Takahashi, Hajime Imamura, Tomohiko Adachi, Hideki Ishimaru, Susumu Eguchi, Hisamitsu Miyaaki

**Affiliations:** 1 Department of Gastroenterology and HepatologyNagasaki University Graduate School of Biomedical SciencesNagasakiNagasaki prefectureJapan; 2 Department of Surgery 200674 Nagasaki University Graduate School of Biomedical SciencesNagasakiNagasaki PrefectureJapan; 3 Department of Radiology 200674 Nagasaki University Graduate School of Biomedical SciencesNagasakiJapan; 4 Department of SurgeryNagasaki University Graduate School of Biomedical SciencesNagasakiJapan

A 50-year-old woman had undergone pancreaticoduodenectomy with hepaticojejunostomy for pancreatic head cancer. Sixteen months later, she developed liver dysfunction and intrahepatic bile duct dilatation, raising the suspicion of a hepaticojejunostomy anastomotic stricture. Local recurrence was excluded by computed tomography, endoscopic assessment, and bile cytology; the stricture was therefore considered benign. Although multi-hole fully covered self-expandable metal stents (FCSEMSs) were designed to maintain side-branch drainage, direct in vivo fluoroscopic documentation of functional bile flow through the side holes remains scarce. This case highlights not only the endoscopic treatment of a benign hepaticojejunostomy anastomotic stricture, but also the functional demonstration of side-branch preservation.

Because of the surgically altered anatomy, double-balloon enteroscopy-assisted endoscopic retrograde cholangiopancreatography (ERCP) was performed. 1​ The anastomosis was reached, and a 7-Fr, 7-cm plastic stent was placed into the left hepatic duct; however, biliary drainage remained insufficient. Liver dysfunction persisted with right intrahepatic duct dilatation and fever. The additional plastic stent placement into the right hepatic duct was attempted but failed because of the severe stricture. Percutaneous transhepatic biliary drainage (PTBD) was therefore established via the segment 5 branch (B5).

**Fig. 1:**
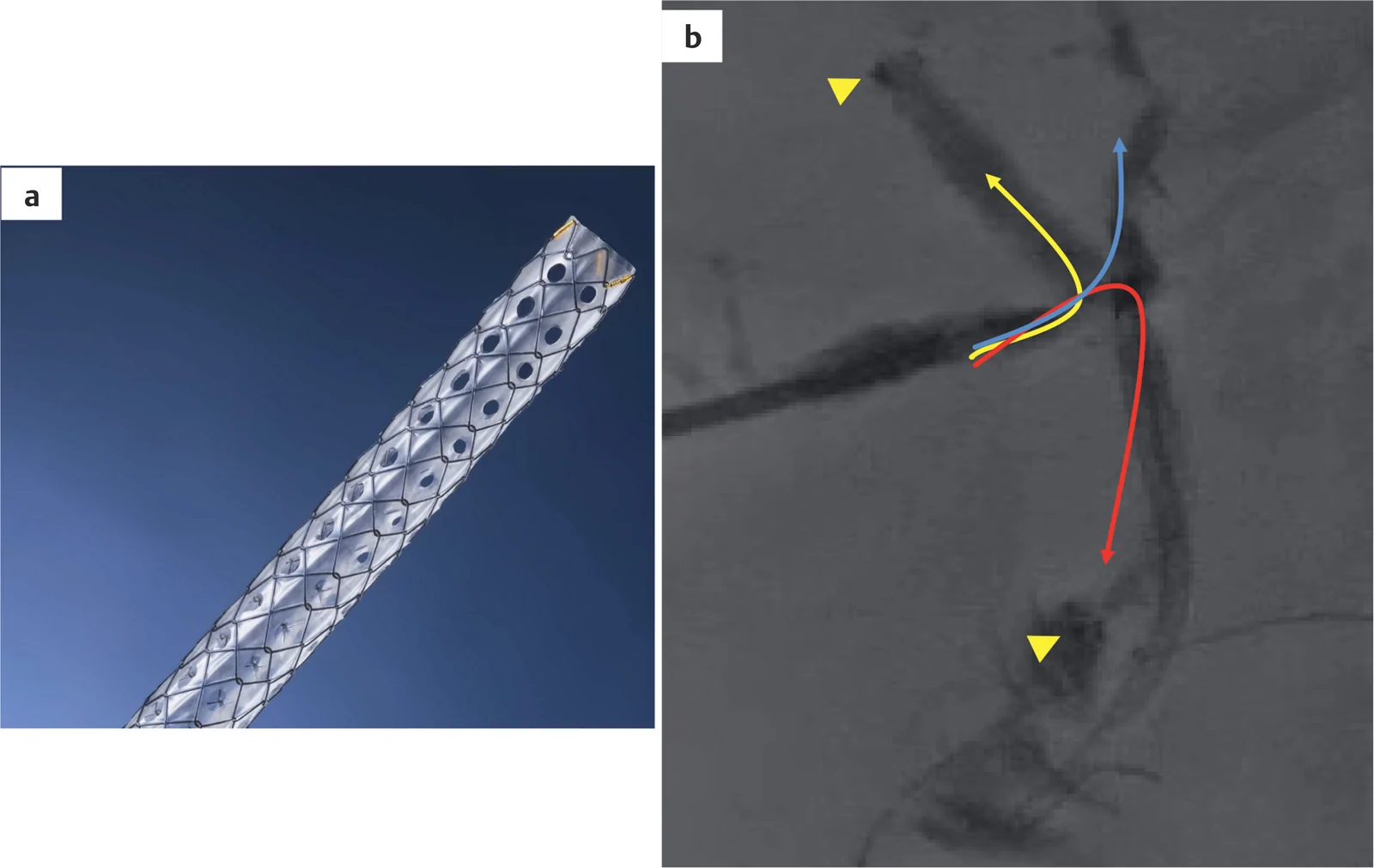
**(a)** A magnified view of the multi-hole fully covered self-expandable metal stent (FCSEMS; HANAROSTENT Biliary Multi-Hole Benefit; 5.9-Fr delivery system) showing the 1.5-mm side holes in the covering membrane. **(b)** A fluoroscopic image: contrast medium injected through the percutaneous transhepatic catheter in the segment 5 branch (B5) passes through the covering membrane (side holes) of the stent and opacifies the segment 8 branch (B8; yellow arrow), the afferent jejunal limb (red arrow), and the left intrahepatic bile duct (blue arrow), demonstrating preserved drainage through the side holes. The yellow arrowheads indicate the proximal and distal ends of the self-expandable metal stent; the semi-transparent blue overlay indicates the multi-hole FCSEMS.

The anastomotic stricture was located immediately below the confluence of the right and left hepatic ducts. Because the 7-Fr plastic stent could not be advanced into the right hepatic duct across this tight stricture, and because a conventional FCSEMS was expected to occlude the adjacent side branch, we selected a multi-hole FCSEMS (HANAROSTENT Biliary Multi-Hole Benefit, 6 mm×60 mm; M.I. Tech Co., Ltd, Seoul, South Korea; distributed by Boston Scientific Japan, Tokyo, Japan). Using double-balloon enteroscopy-assisted ERCP, its thinner 5.9-Fr delivery system was advanced across the stricture that the plastic stent could not traverse, and the stent was deployed into the segment 8 branch (B8) with the side holes facing the B5 orifice ( Fig. 1 ). The previously placed left hepatic duct plastic stent was exchanged for a new 7-Fr plastic stent during the same session. Importantly, contrast medium injected through the indwelling PTBD catheter in B5 was observed flowing through the side holes into the FCSEMS lumen and subsequently into the jejunal limb, providing direct in vivo fluoroscopic evidence that side-branch drainage was preserved ( Video 1 ). The PTBD catheter was subsequently removed, and liver function did not deteriorate during 11 weeks of follow-up.

**Video 1** Contrast medium injected through the percutaneous transhepatic catheter in the segment 5 branch flows through the side holes of the multi-hole FCSEMS into the stent lumen and then into the jejunal limb, providing direct in vivo fluoroscopic evidence of preserved side-branch drainage.


A FCSEMS provides durable radial forces for a refractory anastomotic stricture and can be removed when required; however, its covering membrane may occlude bile duct branches arising along the stented segment. In the present case, the stricture was located immediately below the hepatic confluence, and the conventional FCSEMS placement would have occluded B5, ordinarily necessitating an additional plastic stent to prevent segmental cholangitis. The multi-hole FCSEMS, which was developed mainly for malignant biliary obstruction, addresses this limitation: 1.5-mm side holes in the covering membrane allow the transmembrane drainage of a covered branch, while limited tissue ingrowth through the holes may reduce migration; unlike uncovered self-expandable metal stents, the multi-hole FCSEMS retains removability, making it attractive for benign biliary strictures. The 5.9-Fr delivery system may also facilitate passage through tight strictures. 2​ 3​ ​4​ To our knowledge, the use of a multi-hole FCSEMS for the benign hepaticojejunostomy anastomotic stricture with preservation of side-branch drainage has rarely been described. Moreover, published evidence regarding multi-hole FCSEMSs has mainly focused on technical feasibility and clinical outcomes, whereas direct visualization confirming functional bile drainage through the side holes has rarely been documented. In this patient, contrast flow from the indwelling B5 PTBD catheter through the side holes into the stent lumen was clearly observed, allowing the removal of the percutaneous catheter without additional plastic stent placement and avoidance of long-term external drainage. 5​ This case not only demonstrates the feasibility of multi-hole FCSEMS placement for benign hepaticojejunostomy strictures adjacent to a bile duct branch, but also provides rare in vivo fluoroscopic evidence that the side holes can effectively preserve branch drainage.

Endoscopy_UCTN_Code_TTT_1AO_2AZ
